# Is there a need for a GP consultant at a university hospital?

**DOI:** 10.1186/1471-2296-9-55

**Published:** 2008-09-30

**Authors:** J Mulder, KH Groenier, JH Dekker, AJ Berendsen, J Schuling

**Affiliations:** 1 General Practitioner, t'Z and, The Netherlands; 2 Department of General Practice, University Medical Centre Groningen, University of Groningen, Antonius Deusinglaan 1, 9713 AV Groningen, The Netherlands

## Abstract

**Background:**

Patients in hospital can develop complaints unrelated to the condition they are admitted for. The treating specialist will then call upon a co-specialist who is specialized in the clinical picture associated with the new complaint. For such a complaint, the GP is usually the first contact, when the patient is not in hospital. Normally specialists only encounter patients GPs have selected for referral. The risk of the specialist overestimating the predictive value of 'unselected' complaints and symptoms of a serious condition is high. This may lead to an overuse of diagnostic treatments. Such treatments weigh more heavily on the patient, cause inadequate use of hospital facilities and, as a consequence, generate higher costs.

Because of these considerations, we wished to investigate if there is a need for the GP as a consultant for new complaints during hospital admittance.

**Method:**

The files of a random sample of patients who had an interdisciplinary consultation during their stay in hospital were judged by an expertpanel whether the consultation fitted the expertise of a GP.

**Results:**

In 28 out of 84 files the consultation fitted the expertise of a GP; most cases concerned a specific condition that is not part of the specialist's expertise, most frequently dermatological problems. In a minority of cases the specialist is confronted with a clinical problem with symptoms of which the cause is not clear, for example fever.

**Conclusion:**

Generally, the consultations concern serious, often very complex conditions, i.e. cases that should be assessed by a specialist. Nevertheless, the expert panel's judgment of the interdisciplinary consultations shows that in more than half of the dermatological cases and in a limited number of consultations by a specialist of internal medicine and geriatrics the problems fit the GP's expertise.

Given the morbidity in academic hospitals we suppose that the results of a similar study in a peripheral hospital might even show more perspective for a GP consultant. These results offer sufficient arguments to start a pilotstudy into the role of a GP consultant in hospital.

## Background

The role of the GP in hospitals varies greatly in different countries. In the United States, for example, the GP has a place in small, general hospitals. In Canada GPs have a few beds reserved where they can admit their own patients and call for a specialist consultation. In the United Kingdom, GPs have gained experience in the hospital emergency department. It has been shown that hospital doctors and medical specialists ask more often for additional tests than GPs in the emergency setting [1]. In the United Kingdom, GPs with a special interest have also been employed, who carry out substituting tasks for patients of certain categories [2].

The Netherlands have traditionally made a strict division between family medicine and specialist care. If needed, GPs refer patients to a specialist. Should the specialist choose to have the patient admitted, he is responsible for the care during admittance.

Sometimes, hospitalized patients develop a symptom that is unrelated to the condition they are admitted for. The treating specialist will then call upon a co-specialist who is specialized in the clinical picture associated with the new symptom, so he can help with diagnosis and therapy. However, the new symptom may be quite common, concerning for example the musculoskeletal system, upper respiratory tract infections, or cutaneous diseases. For such a complaint, the GP is usually the first contact, when the patient is not in hospital. Normally specialists only encounter patients GPs have selected for referral. The morbidity pattern a specialist's meets, is very different from that of a GP especially in a university hospital. The risk of the specialist overestimating the predictive value of 'unselected' complaints and symptoms of a serious condition is high. Should a specialist be consulted for problems usually dealt with by the GP, this may lead to a rise in diagnostic procedures. Such procedures weigh more heavily on the patient, cause inadequate use of hospital facilities and, as a consequence, generate higher costs.

Because of these considerations, we wished to investigate if there is a need for the GP as a consultant for new symptoms during stay in hospital.

We formulated the following research questions:

1. How many patients admitted at the university hospital need consultation for a symptom other than the one they were admitted for?

2. What is the nature of these consultation requests and are they sufficiently clear?

3. Which requests fit the GPs' expertise?

## Method

We decided upon a retrospective study of patients who had an interdisciplinary consultation during their stay at the University Medical Centre of Groningen (UMCG). In order to formulate possible inclusion criteria for a sample we searched Pubmed ('95 until present) with the MESH terms 'referral and consultation' and with the free text 'interdisciplinary consultation'. This search, however, yielded no articles that concern the topic of this study so that no criteria for the sample could be formulated.

We used the medical financial administration of the hospital to retrieve all patients that had an interdisciplinary consultation during a hospital stay in 2006. Patients who were admitted to the department of psychiatry were excluded.

Consecutively two random samples were drawn. With the data of the first sample we wanted to answer to the first and second study question. We decided that if a GP could play a role as a consultant in at least 25% of the consultations of a certain discipline, that discipline would be included in the second part of the study to give an answer to the second and third question. Based on this criterion, the second sample was limited to three consulted disciplines (dermatology, internal medicine and geriatrics).

The GP researcher went through the dossiers, and registered the following relevant data on a form:

### Patient data for interdisciplinary consultations

UMCG number

Date of birth

Age

Gender

Length of admittance

Date of consultation

Admitting department/discipline requesting consultation

Consulted discipline

Patient known to discipline

Follow-up consultations

Reason for admittance

Co morbidity

Consultation request

Quantity of the request

Quality of the request

Advice consultant/interventions

Diagnosis consultant

Discharge diagnosis

Clarity on follow-up

Reporting back to the GP

A panel of experts judged the consultations (as registered by the GP researcher). The choice for an expert panel was made because no criteria could be derived from the literature, i.e. the experience based knowledge of the members determined the ultimate judgment. This panel consisted of four members: an experienced GP with an academic background, an experienced GP form a peripheral practice, a general internist and a clinical geriatrist (both working in an academic setting). We chose these last disciplines because of the broad scope of their expertise.

The panel members had three options to choose from in judging the consultations:

1. Yes, this problem fits the expertise of the GP.

2. There is doubt this problem would fit the expertise of the GP.

3. No, this problem does not fit the expertise of the GP.

It was also judged whether the registered consultation request was well-defined and whether the relevant discipline had been contacted before. As well, it was determined if the end report was clear on the follow-up of the consultation.

The panel members read and evaluated all the registration forms. Subsequently, the panel members discussed all cases in two sessions lead by the project leader, during which the researcher provided additional information from the dossiers when needed or wanted. When the panel members disagreed, they discussed the case until consensus was reached.

## Results

According to the medical financial administration out of all the patients admitted at the UMCG in 2006 (about 32000) an interdisciplinary consultation was requested for 2257 patients (9%). Those admitted to the department of psychiatry were excluded (41), leaving 2216 dossiers (table 1) out of which a first random sample of 60 dossiers was drawn.

**Table 1:** Number of interdisciplinary consultations at UMCG in 2006

Discipline	Consultations	%
Dermatology	878	31
Surgery	333	12
ENT	314	11
Oral surgery	270	10
Paediatric neurology	158	6
Ophthalmology	144	5
Internal medicine	119	4

In 25 dossiers there was no record of the interdisciplinary consultation, leaving 35 dossiers for analysis.

An interdisciplinary consultation is requested most often for patients staying at the departments of internal medicine, surgery and neurology. This spread is consistent with the total number of admissions for these disciplines in 2006.

The average length of admittance for all admitted patients is 10 days. For admitted patients who had an interdisciplinary consultation, this is 20 days. The longer the hospital stay, the higher the chance of an interdisciplinary consultation. Both genders were equally represented (50-50) in the patients.

Consultation is given to all ages, though there are clearly more consultation requests for patients ageing between 59 and 70 years old.

In the first sample of the 35 randomly selected dossiers three of the consulted disciplines met the 25% criterion: dermatology, general internal medicine and geriatrics.(table 2)

**Table 2:** Cases from the first random sample (n = 35)

Consult requesting discipline	Diagnosis	Consulted discipline	Question/diagnosis	Judgment expert panel: yes/no to GP
Internal medicine	Fever e.c.i.	Orthopaedia	Infected hip prosthetic	No
	Geriatric issues	Psychiatry	Psychiatric diagnosis	No
	Colitis ulcerosa	Gynaecology	Check during pregnancy	No
	Liver cirrhosis	Dermatology	Treatment lice	Yes
	Multiple issues	Dermatology	Treatment lip oedema	Yes
	AML, hepatorenal syndrome	Dermatology	Treatment oedema	No
	Respiratory insufficiency	Gynaecology	Judgment	No
	Lymph node metastases	Gynaecology	magnesium suppletion	No
	M. Crohn, liver transplant	Dermatology	Gynaecological primary tumor	Yes
	Hypertension, complex issues	ENT		No
		Gynaecology	Treatment wart	No
	Cardial asthma	Dermatology	Deviation sinus, tumor	Yes
	Disturbance of consciousness	Geriatrics	Post menopausal blood loss	No
	Arhythmia, delirium	Cardiology	Hypostatic eczema	No
	Osteosarcoma	Urology	Judgment delirium	No
	Joint complaints	Psychiatry	Cardial ischemia	No
	Disturbance of consciousness	Dermatology	Bladder pathology	Doubt
	Systemic disease	Psychiatry	Conversion of schizophrenia	No
	Liver cirrhosis	Psychiatry	Paronychia	No
	Neuroleptic malignant		Psychiatric screening	
	syndrome		Co treatment	
Surgery	Oesophageal carcinoma	ENT	Recurrent paresis	No
	Rectal carcinoma	Neurology	CVA	No
	Stomach ache, no surgical	Gynaecology	Gynaecological cause	Doubt
	deviation	Ophtalmology	Screening diabetic	No
	Tendon sheath panaritium	Cardiology	retinopathy	No
	Bronchial carcinoma		Decompensation cordis	
Neurology	Transverse lesion	Urology	Treatment urosepsis	No
	Tumor cerebri	ENT	Judgment infection focus	No
	Polytraumatised	Jaw surgery	Judgment fracture recovery	No
	CVA	Ophtalmology	Funduscopy embolies	No
	CVA	ENT	CT-deviation throat and thorax	No
	CVA	Dermatology	Bandaging DVT	Yes
	CVA	Internal medicine	Diabetes mellitus and dehydration	No
Cardiology	Intended valve replacement	Oral surgery	Judgment infection focus	No
Gynaecology	Vulvar carcinoma	Dermatology	Bandaging lymph node oedema	Yes
Paediatrics	Benign brain tumor	Paediatric neurology	Increased intracranial pressure	No

In the second sample of 67 dossiers restricted to these three disciplines 18 dossiers had no record of the interdisciplinary consultation, leaving 49 dossiers for analysis. The results are summarized in table 3.

**Table 3:** Cases from the second random sample (n = 49)

Consult requesting discipline	Diagnosis	Consulted discipline	Question/diagnosis	Judgment expert panel: yes/no to GP
Paediatrics	Lymph node oedema	Dermatology	Treatment wart	Yes
	Neurological issues	Dermatology	Treatment monoliasis	Yes
	Muscular dystrophy, cardiomyopathy	Dermatology	Seborrhoic eczema	Yes
	Extended hemangioma	Dermatology	PHACES syndrome	No
Neurology	Transverse lesion	Internal medicine	Fever e.c.i.	No
	Impaired awareness		Delirium, complex issues	No
	Impaired awareness	Geriatrics		No
	Cerebral deviations, artificial respiration	Geriatrics Internal	Delirium urinary tract infection	No
	Suspicion of ALS	Medicine	Fever and dehydration	
		Dermatology	Toxicodermia	
Surgery	Pancreatoduodenectomy	Dermatology	Treatment herpes zoster	Yes
	Abdominal aortic aneurysm	Dermatology	Herpes simplex infection	Yes
	Septic embolies	Internal medicine	Fasciitis necroticans	No Doubt
	Arteriosclerotic amputation	Geriatrics	Delirium	No
			Generalised oedema	Doubt
	Pancreatitis	Dermatology	Pitting oedema, decubitus	Yes
	Polytraumatised	Dermatology	Bone densimetry	No
	Ankle fracture	medicine	VAC treatment	
	Non-healing leg wound	Dermatology		
Urology	Stone removal	Dermatology	Treatment herpes simplex	Yes
Gynaecology	Vulvar carcinoma	Dermatology	Treatment erysipelas	No
Internal	Fever e.c.i.	Dermatology	Treatment ulcera cruris	Doubt
medicine	Heart failure	Dermatology	Treatment ulcera cruris	Yes
	Heart failure	Geriatrics	Treatment delirium	Yes
	Oedema alcohol abuse	Dermatology	Treatment oedema, dry skin	Yes
	Colotis ulcerosa, pneumonia	Dermatology		Yes
	M. Kahler, sepsis	Dermatology	Judgment pustulous	No
	Asthma, adipositas	Dermatology	condition	Doubt
	Heart failure	Geriatrics	Bullous erysepilas	No
	Kidney biopsy	Dermatology	Erysepilas	Yes
	Atrial fibrillation, diabetes	Geriatrics	Delirium, complex issues	Doubt
	mellitus	Dermatology		Yes
	Liver cirrhosis	Dermatology	Oedema without DVT	Yes
	Pneumonia	Dermatology	Delirium	No
	Bacteremia streptococci	Dermatology	Oedema	No
	Bacteremia streptococci	Dermatology	Herpes simplex infection	No
	Erysipelas	Dermatology	Toxicodermia	Yes
	Intestine issues	Dermatology	Decubitus	No
	Sepsis	Dermatology	Orthoergic eczema	Yes
	Pancreatitis	Dermatology	Pitting oedema	No
	Vasculitis	Dermatology	Bullous erysepilas	No
	T-cell lymphoma	Dermatology	Xerosis cutis	Yes
	Ascites peritonitis sclerosis	Dermatology	Ulcera lower legs	Yes
		Geriatrics	Panniculitis treatment	No
	Kidney insufficiency	Dermatology	Pitting oedema	No
	Collaps, dyspnoe d'effort	Dermatology	Xerosis cutis	Yes
	T-cell lymphoma, sarcoidosis		Delirium	
	Pancreatitis		T-cell lymphoma pleuritis Pitting oedema	
Pulmonary diseases	Respiratory insufficiency	Dermatology	Eczema	Yes
Orthopaedia	Total hip prosthetic	Geriatrics	TIA	No
ENT	Mastodoitis, facial paresis	Internal medicine	Pancytopenic fever	No

During the consensus meeting, the members of the expert panel came to the following conclusions concerning the consultation requests:

The requests were mostly concise and the panel judged 60% of the cases as qualitatively good. In most cases, the report of the consulted specialist was judged as adequate.(3)

In 60% of the cases, the specialist made an appointment for a follow-up consultation. What the follow-up would entail was nearly always clear.

In 23% of the cases, the GP was not informed about the consultation at discharge of the patient.

In 94% of the cases, the expert panel reached consensus on whether or not the GP could have conducted the concerned consultation. Out of 82 cases, the panel found that 28 cases could have been handled by the GP versus 48 cases which could not have been. In 6 cases, the panel was not sure. (table 4) For most cases, the expert panel's judgment was unanimous. In 5 cases, the ratio was 3 against 1.

**Table 4:** Judgment from the expert panel on the interdisciplinary consultations (N = 84)

Consensus expert panel:	
GP consultant good alternative	28
GP consultant no alternative	48
Doubt	6
No consensus expert panel	2

## Discussion

Although the expert panel judged two third of the consultation requests as good, they agreed that many of the specialists did not formulate a clear request, but instead made a list of the observed problems and asked for treatment advice [3]. Especially for complex problems, the specialist does not seem to judge carefully beforehand which discipline would be best suited, based on priorities. In such cases, the discipline receiving the consultation request appeared to choose a solution based on the options the discipline could offer.

In cases where the expert panel could not reach consensus or took a long time to do so, this had to do with insufficient information on the medical history of the patient.

The level of expertise of the doctor requesting consultation was also considered to be of importance to the question of whether the GP could have conducted the consultation or not. Judging the general knowledge of an internist asking a geriatrist for a consultation on complex issues, for example, consultation by a GP seems less obvious.

In a small number of cases GPs and specialists of the panel could not reach agreement on the possible role of the GP.

Most cases of interdisciplinary consultation concern a specific condition that is not part of the specialist's expertise, for example dermatological problems. Skin conditions that can be treated by the GP according to the committee, are viral infections concerning the herpes simplex virus and the varicella zoster virus, venous ulcera cruris, venous insufficiency oedema, and superficial skin conditions concerning bacteria, fungi and yeast. Also eczema and xerosis cutis are conditions that can be treated by the GP.

In a minority of cases the specialist is confronted with a clinical problem with symptoms of which the cause is not clear, for example fever.

Generally, the consultations concern serious, often very complex conditions, i.e. cases that should be assessed by a specialist. Nevertheless, the expert panel's judgment of the interdisciplinary consultations shows that in more than half of the dermatological cases and in a limited number of consultations by a specialist of internal medicine and geriatrics the problems fit the GP's expertise. On a total number of 878 dermatological consults this implies a potential number of 527 consults for a GP consultant yearly in this discipline alone.

Furthermore both samples of interdisciplinary consults had a considerable number of missing reports (40% and 25% resp), which should be taken into account when estimating the potential workload for a GP consultant.

With regard to the grave and complex problems of patients in academic hospitals we suppose that the results of a similar study in a peripheral hospital might even show more perspective for a GP consultant.

## Conclusion

This study focused on the possible role of a GP consultant at the hospital, concerning admitted patients suffering from a condition other than the one they were admitted for. Our research shows there might be such a role for a GP, especially for dermatological problems. Our results offer sufficient arguments to start a pilot study on the role of a GP consultant in a hospital. Such a project would learn us more about the acceptance of a GP consultant by specialists and may open up possibilities for a new role: one in which the GP gives advice on choice-making, prioritizing, and determining the applicability of treatment plans.

## Appendix

### Examples of an interdisciplinary consultation that could have been conducted by a GP

A 20 year-old man is well-known to M. Crohn and admitted at internal medicine after a liver transplant. The immunosuppressive therapy given because of the transplant causes warts, for which a dermatologist is called in. The dermatologist's diagnosis is verrucae vulgares and prescribes liquid N2 for the face and monochloric acid for the hands. (17)

A 56 year-old woman is admitted to internal medicine because of a decompensated liver cirrhosis due to alcohol abuse. A psychiatrist is asked to confirm the alcohol abuse. No cognitive problems or psychopathology are found, though the patient possibly leans towards an avoidance personality. In the mean time, the alcohol abuse has ceased resolutely. (36)

### Example of an interdisciplinary consultation that could not have been conducted by a GP

An 82 year-old man is admitted to internal medicine, experiencing blackouts. His dossier shows he ahs had a CVA and suffers from cardiac arhythmia. He has developed a progressive dyspnoe d'effort, disorientation and a reversal of day and night rhythm. A geriatrist was asked if this could be a case of a delirium. A Cheyne Stokes respiration pattern is diagnosed, as a consequence of cerebral damage and behavioural changes due to the CVA. Based on these problems and the hospital admittance, a delirium has developed, for which the patient receives medicinal treatment and nursing advice. (25)

### Example of doubt

A 72 year-old is admitted to the geriatric department for observation of geriatric problems. His medical history shows angina pectoris, status post CABG, COPD and kidney insufficiency. A psychiatrist is asked to evaluate the possibility of an affective and/or a personality disorder. A depressive disorder, on top of a long history of recurring depressions is diagnosed, as well as a probable pervasive disorder of the Asperger type. The psychiatrist advices to put the patient on citalopram. (04)

## Competing interests

The authors declare that they have no competing interests.

## Authors' contributions

All authors contributed to the design and write-up of this study. JM carried out the data collection and wrote the first draft of the report. KHG advised on the study design and data analysis. JD and AJB commented on several versions of the manuscript, and JS was projectleader, and chairman of the expertpanel. All authors read and approved the final manuscript.

## Pre-publication history

The pre-publication history for this paper can be accessed here:


